# Methane emissions reduced using gypsum in pilot‐scale dairy manure tanks

**DOI:** 10.1002/jeq2.70112

**Published:** 2025-12-28

**Authors:** Emma Tomalty, Mélodie Laniel, Vyncent Leblanc, Ulrica McKim, Sandra Yanni, Patricia Moher Copa, John McCabe, Robert Gordon, Andrew VanderZaag

**Affiliations:** ^1^ Agriculture and Agri‐Food Canada, Science and Technology Branch Ottawa Ontario Canada; ^2^ Nova Scotia Department of Agriculture Bible Hill Nova Scotia Canada; ^3^ School of the Environment University of Windsor Windsor Ontario Canada

## Abstract

Animal manure storage facilities are sources of greenhouse gas emissions in Canada, with the majority of emissions in the form of methane and nitrous oxide. This study was conducted to assess how effective adding gypsum powder to cow manure is at reducing the emissions of methane and nitrous oxide. At a pilot‐scale research facility in Nova Scotia, Canada, gypsum powder was added to tanks containing ∼10,000 L of local dairy manure in duplicate at three rates: low rate (3.2 g L^−1^), high rate (7.1 g L^−1^), and the control (0 g L^−1^). Each manure tank was enclosed within a steady‐state chamber, and gas samples were manually taken using a syringe from the exhaust into pre‐evacuated vials at regular intervals between June and November (143 days). Using gas chromatography, methane and nitrous oxide measurements were analyzed from each vial, allowing for calculations of cumulative emissions and CO_2_‐e. Compared to the control, both the high rate and low rate of gypsum significantly reduced cumulative methane emissions. Though the cumulative nitrous oxide emissions were reduced using both rates of gypsum, the nitrous oxide emissions were minor and not statistically significant. Methane emissions were reduced by 87% using the low rate of gypsum and by 92% using the high rates of gypsum. Gypsum additive to slurry in storage facilities is a safe and effective mitigation strategy to reduce greenhouse gas emissions. Further research is required to support on‐farm application rates and feasibility.

## Core Ideas

- Gypsum was added to dairy slurry stored for 143 days in a pilot‐scale study (10,560 L slurry tanks).
- Gypsum added at high (7.1 g L^−1^) and low (3.2 g L^−1^) rates significantly reduced CH_4_ emissions compared to control.
- Methane emissions were reduced by 92% and 87% using the low and high rates, respectively, compared to the control.
- Adding gypsum did not change the pH or total ammoniacal N content of the manure.
- Total greenhouse gas emissions (CO_2_‐e) were reduced by 91% and 85% for high and low rates, respectively, compared to control.

## Plain Language Summary

Canadian farmers store dairy manure in outdoor tanks that are open to the air. There are processes that happen within the manure that release toxic gases called greenhouse gases (GHGs) into the air that are harmful to the Earth's atmosphere, such as methane and nitrous oxide. The purpose of this research was to determine if adding gypsum, a naturally occurring material commonly used in drywall, to dairy manure would reduce the release of GHGs. This was done by measuring the GHGs released from six different outdoor tanks filled with dairy manure and three different rates of gypsum (low, high, and zero). The results showed that the high and low rates of gypsum reduced the release of both GHGs but worked better at reducing the release of methane. This is important because methane is a more potent GHG than nitrous oxide, so a reduction in methane means an overall reduction in GHGs released from the dairy manure.

**Abbreviations**
DFC: Dairy Farmers of Canada
FAO: Food and Agriculture Organization
FOS: fatty‐acid content
GHG: greenhouse gas
HSD: honestly significant difference
IPCC: Intergovernmental Panel on Climate Change
LCA: life cycle analysis
MCF: methane conversion factor
ORP: oxidation‐reduction potential
TAC: buffering capacity
TAN: total ammoniacal nitrogen
TN: total nitrogen
TS: total solids
VS: volatile solids

## 1 INTRODUCTION

Greenhouse gas (GHG) emissions come from both natural and anthropogenic sources. Some of the biggest anthropogenic sources contributing to GHG emissions are fossil fuel production, the waste industry, and the agriculture industry (Malley et al., 2023). Global efforts toward decarbonization and climate change mitigation in the form of initiatives and agreements are ongoing. For instance, the 2015 Paris Agreement, signed by 195 countries, aims to limit the increase in global temperature to under 1.5°C (United Nations, 2015). Methane (CH_4_), an important GHG, has a short atmospheric lifetime and a high global warming potential, meaning that the impact of CH_4_ emissions is especially strong in the near term (Filonchyk et al., 2024). Consequently, efforts focused on limiting CH_4_ emissions are beneficial at slowing global warming in the near term while benefiting long‐term climate change goals. This is reflected in the United Nations Global Methane Pledge, signed by 149 countries, with one of the primary goals being a 30% reduction of 2020 level CH_4_ emissions by 2030 (Cael & Goodwin, 2023). Globally, the agriculture industry accounts for 22% of total GHG emissions and approximately 40% of total CH_4_ emissions (Crippa et al., 2021; IPCC, 2021). Within Canada, a mostly temperate climate, the agriculture industry contributes 10% of total GHG emissions and about 35% of total CH_4_ emissions, with livestock production being a key contributor (Environment and Climate Change Canada, 2024). To complement global efforts, Canada‐specific initiatives have been launched as well, including the Dairy Farmers of Canada (DFC) goal to reach net‐zero emissions from dairy production by 2050 through mitigation practices that reduce emissions (Dairy Farmers of Canada, 2024). Achieving the targets set by the DFC requires a variety of options to meet the farmers’ needs, due to varying farm sizes and objectives, and to encourage broader adoption of mitigation strategies.

Dairy manure is rich in carbon and nutrients, making it a valuable soil fertilizer (Baldé et al., 2016; Dalby et al., 2021). A common practice by dairy farmers across the globe is to store their manure, often in outdoor, uncovered, liquid manure (slurry) storages, so that it can be used to fertilize their cropland. In temperate climate regions, like Canada and many places in northern Europe, emissions are generally limited to the warm season (June to November) (Balde et al., 2016; Cardenas et al., 2021; Im et al., 2020). The conditions within slurry storages facilitate production and emission of CH_4_ and nitrous oxide (N_2_O) (Cardenas et al., 2021). Nitrous oxide is a concerning GHG due to its high 100‐year global warming potential 273 × higher than CO_2_ (IPCC, 2021). However, N_2_O emissions from liquid storage tend to be a small proportion of total GHG emissions (Sokolov et al., 2021, 2019). Methane emissions are a larger concern since outdoor manure storage contributes approximately 5% of global CH_4_ emissions (FAO, 2022). Due to the short atmospheric lifetime and high global warming potential of CH_4_, 27 × that of CO_2_, CH_4_ is often referred to as the most important non‐CO_2_ GHG associated with livestock production (Ambrose et al., 2023; IPCC, 2021).

Mitigation strategies to reduce CH_4_ emissions from slurry storage include both physical and chemical strategies, some of which are already being implemented and investigated at full scale in Europe (Flotats et al., 2011; Hjorth et al., 2010; Kai et al., 2008; Lemes et al., 2022; Nisbet et al., 2025; Vechi et al., 2023). Physical strategies have been the focus of many pilot‐scale and lab‐scale studies in recent decades, including gas‐collection covers, solid‐liquid separation, and manure aeration (Amon et al., 2006; Holly et al., 2015; VanderZaag et al., 2018, 2010; Vechi et al., 2023). Methane production in slurry is largely driven by anaerobic microbes (i.e., methanogens), which break down organic matter; therefore, chemical strategies that inhibit methanogenesis is an area of considerable research interest. A recognized chemical strategy is slurry acidification, commonly used in countries in Europe to reduce ammonia (NH_3_) volatilization from slurry storage (Finzi et al., 2024).  Slurry acidification inhibits methanogenesis by lowering the pH leading to less active methanogens and increasing competition with sulfate reducing bacteria (Ambrose et al., 2023). A pilot‐scale study on slurry acidification revealed that low doses of sulfuric acid (H_2_SO_4_) during the warm season was effective at reducing CH_4_ emissions by 87% (Sokolov et al., 2019). Similar results from acidified manure were found in a farm‐scale study investigating emissions from pig farms with different manure management practices (Vechi et al., 2022). Co‐benefits of lower pH are reduced NH_3_ volatilization and increased sulfur content, thereby improving the manure fertilizer value (Sokolov et al., 2021). However, it has been observed that the benefits of competition with sulfate reducing bacteria may be inhibited when the slurry is acidified to pH < 5.5 (Dalby et al., 2021). The complexity of maintaining optimal slurry pH and handling acid on farms may pose a challenge to rapid, voluntary adoption by Canadian farmers. sulfate addition changes the microbial community by effectively utilizing hydrogen, inhibiting methanogen production, and enhancing the bacteria that utilize sulfate. This in turn effectively limits methane production (Petersen et al., 2012). It has been proposed that sulfate availability may be the primary influencing factor, rather than pH, in the process of reducing CH_4_ emissions based on studies comparing different acids and sulfate containing chemicals (Eriksen et al., 2012; Matos Pereira Lima et al., 2025a).

Gypsum (CaSO_4_) powder has been suggested as an alternative mitigation strategy by promoting sulfate reducing bacteria to increase competition for substrates without lowering the slurry pH. Recent research on the effects of various chemical additives on methane emissions from slurry shows that a high dose (7.3 g L^−1^) of gypsum additive resulted in 42% reduction in CH_4_ emissions, comparable to the 37% reduction achieved with a low dose of sulfuric acid additive after 80 days of incubation at 24°C (Matos Pereira Lima et al., 2025a). A follow‐up study showed greater reductions over a longer time at lower temperatures with a lower dose of 2.0 g L^−1^ gypsum (e.g., 57.6% at 18°C, 56.2% at 21°C; Matos Pereira Lima et al., 2025b). These results support an earlier laboratory study on the effects of gypsum additive to liquid swine manure, resulting in a reduction of CH_4_ emissions by 50% with 4% w/w gypsum (Berg & Model, 2008). A laboratory study compared the efficacy of a commercial additive made up of 100% agricultural gypsum (SOP Lagoon) and locally sourced gypsum, which showed a similar reduction in CH_4_ emissions at similar application rates (Sauvé et al., 2025). This study demonstrated that locally sourced gypsum was an effective, low‐cost alternative to commercial additive products; however, the experiment used anaerobic bottles under constant temperatures, highlighting a need to study the effects of gypsum at a larger scale. A recent study assessed the efficacy of eight different slurry additives in reducing methane emissions in either a meso‐scale experiment using 20 L of slurry in duplicate for 92 days in winter conditions (average ambient temperature of 6°C) or a pilot‐scale experiment using 660 L of slurry in triplicate for 77 days in winter conditions (9°C) (Owusu‐Twum et al., 2024). These experiments revealed that all the sulfate containing additives, including gypsum and lactogypsum, reduced CH_4_ emissions by 46%–87% using doses ranging from 25 to 100 g L^−1^ (Owusu‐Twum et al., 2024). However, to date, studies on gypsum additives have only been done under constant temperatures at laboratory scale or under winter conditions at pilot scale. There is a need to evaluate this approach where manure is stored outdoors during summer conditions and exposed to seasonal temperature variations.

This study addresses the need to test the CH_4_ mitigation potential of gypsum at a larger scale. Three treatments of gypsum additive (zero, low, and high rates) were studied at a pilot‐scale research facility in Nova Scotia, Canada. The objectives of this research were to understand the effects of gypsum additives on cumulative warm‐season GHG emissions from dairy manure. This is important to determine if gypsum can be a cost‐effective and low‐risk alternative compared to acid additives, which may enable widespread adoption on dairy farms.

**Core Ideas**

- Gypsum was added to dairy slurry stored for 143 days in a pilot‐scale study (10,560 L slurry tanks).
- Gypsum added at high (7.1 g L^−1^) and low (3.2 g L^−1^) rates significantly reduced CH_4_ emissions compared to control.
- Methane emissions were reduced by 92% and 87% using the low and high rates, respectively, compared to the control.
- Adding gypsum did not change the pH or total ammoniacal N content of the manure.
- Total greenhouse gas emissions (CO_2_‐e) were reduced by 91% and 85% for high and low rates, respectively, compared to control.

## 2 MATERIALS AND METHODS

### 2.1 Site description and manure additives

The study was conducted at a research site located at Dalhousie University's Bio‐Environmental Engineering Centre in Bible Hill, Nova Scotia (45°45′ N, 62°50′ W). The site contains 6 individual, in‐ground manure storage tanks located outdoors. Each tank (6.6 m^2^, 11.88 m^3^) is enclosed under chambers made of greenhouse plastic with aluminum frames that allowed continuous air flow‐through systems to simulate a steady‐state, enclosed environment. Additional details on this manure storage system have been previously described by VanderZaag et al. (2010) and Wood et al. (2012). Continuous air flow within the chamber, ∼0.5 m^3^ s^−1^ from inlet opening to exhaust fan, allowed for laminar air flow with about two full air exchanges per minute (Le Riche et al., 2017; Wood et al., 2012). Air speed was measured using continuous cup anemometers (m s^−1^) (Weather Monitor II, Davis Instruments) within the venturi outflow ducts of each tank connected to CR10X datalogger (Campbell Scientific) to enable automated recording of 1‐min averages.

The six tanks were emptied and cleaned prior to the start of the experiment and then filled with liquid dairy manure (June 6, 2024) to a depth of ∼1.6 m for 10.56 m^3^ of manure. Manure was obtained from a commercial dairy farm in Shubenacadie, Nova Scotia (45°06′ N, 63°23′ W), which milks ∼120 cows in a facility that uses wood shavings as bedding material.

Industrial‐grade gypsum powder (CaSO_4_·H_2_O, minimum 80% purity) was obtained from Mosher Limestone Company Limited in Upper Musquodoboit, Nova Scotia, in 18 kg bags. The gypsum and manure were added to each tank simultaneously on June 6, 2024, and then stored until November 24, 2024. The tanks were divided into two blocks with three tanks each. The experimental treatment for each block included three rates of gypsum addition: low (3.30 g L^−1^), high (7.26 g L^−1^), and the control (0 g L^−1^). Assuming manure produced by each cow in a barn is 60 L day^−1^, the amount of gypsum added per L of manure ranged from 2.3 to 56 g L^−1^, which equates to approximately 192 g cow^−1^ day^−1^ (or 0.4 lb cow^−1^ day^−1^) and 426 g cow^−1^ day^−1^ (0.9 lb cow^−1^ day^−1^) for the low and high rates, respectively. The low and high rates of gypsum addition were determined from the results of preliminary laboratory studies evaluating CH_4_ suppression using gypsum at different rates and temperatures (Matos Pereira Lima et al., 2025a, 2025b).

### 2.2 Environmental measurements

Manure samples from each tank were collected five times throughout the experiment: June 6, August 9, September 11, October 31, and November 24, 2024. For each tank, a composite sample consisted of samples from three horizontal locations and 3 depths (nine in total), collected using a long sampling rod with a manually operated opening to prevent mixing between sampling locations. Each composite sample was homogenized in a 20 L pail, then divided into three 1000 mL subsamples, and frozen before analysis. One set of subsamples from June 6, August 9, September 11, and November 24, was analyzed for total solids (TSs), volatile solids (VSs), pH, total carbon, total nitrogen (TN), total ammonium nitrogen, and total sulfur at the Nova Scotia Department of Agriculture's Provincial Soils Laboratory in Bible Hill, NS. One set of subsamples for all sampling dates was analyzed for TS, VS, pH, oxidation‐reduction potential (ORP), fatty‐acid content (FOS), and buffering capacity (TAC) at Agriculture and Agri‐Food Canada in Ottawa, ON. Sulfate, sulfur, and analysis were performed on one set of subsamples for each sampling date, and fiber analysis was performed on the samples taken on June 6, 2024, at A&L Laboratories, London, ON.

Temperature measurements within each tank were recorded every 60 min using a combined temperature sensor and Data Logger (E348‐MX2201 HOBO Pendant MX Water Temperature Data Logger; Onset HOBO). Chamber air temperatures were measured at 20 cm above the manure surface inside a radiation shield. Manure temperatures were taken 80 cm below the manure surface at approximately the middle of the tank. Ambient air temperature measurements were measured using a shielded thermistor at two points, 1.7 m above the ground and 0.3 m in front of two of the tanks (E348‐MX2302A HOBO External Temperature/RH Sensor Data Logger; Onset HOBO).

Water was added to each tank using a garden hose seven times throughout the experiment (July 13, July 21, August 4, September 2, October 1, October 2, and November 1, 2024) to simulate rainfall and to maintain a constant volume of manure within each tank.

### 2.3 Gas measurements and flux calculations

Triplicate 20 mL gas samples were manually taken at two ambient locations and at each exhaust duct approximately three times per week. The first and last day of gas sample collections were June 23 and November 12, 2024, respectively (143 days). The gas samples were collected in a syringe and were transferred into 12 mL pre‐evacuated septum‐capped glass vials (Labco Ltd.). To prevent the contamination of outside gases in the event of a leak, the vials were over‐pressurized. Ambient air was sampled at the site of ambient air temperature measurements and was assumed to represent the inlet air of all chambers. The vials were transferred to Agriculture and Agri‐Food Canada, Ottawa, ON, for analysis of CH_4_ and N_2_O using a gas chromatograph (7890B; Agilent Technologies). Flux calculations were performed using the steady‐state equation (Livingston & Hutchinson, 1995):

(1) $$F=\frac{Q}{A}\left(C_{0}-C_{i}\right)$$

Where F is the flux density (mg m^−2^ s^−1^), Q is the airflow rate (airspeed in the exhaust duct × cross‐sectional area of the exhaust duct, m^3^ s^−1^), A is the surface area of the manure tank (m^2^), $C_{0}$ is the gas concentration in the outlet air (mg m^−3^), and $C_{i}$ is the gas concentration in the inlet air (mg m^−3^).

Hydrogen sulfide (H_2_S) gas was measured once approximately halfway through the study period (August 30, 2024) using a multi‐gas detector (G450, GFG Instrumentation, Inc.) in ambient air and inserted into the exhaust duct of each tank to measure the concentration for at least 15 min.

### 2.4 Data analysis

In total there were 52 days out of the 143‐days study period with gas samples. A linear interpolation was performed to obtain a complete dataset for the study period between the first and last day of gas sample collection. Due to precision issues with the gas chromatograph's N_2_O measurements, the last 5 days of the study were not able to be measured, and instead concentrations were assumed to be equal to the average of the previous 5 days. Cumulative emissions of CH_4_ and N_2_O were calculated by summing the total in each tank over the 143‐days interpolated dataset.

The methane conversion factor (MCF) was calculated for each tank using the VS measurements obtained from the manure samples collected June 6, 2024, and maximum potential CH_4_ production (*B*_0_ = 0.24 m^3^ CH_4_ kg^−1^) following the IPCC protocol (IPCC, 2019, 2013). The CO_2_‐e were calculated by multiplying the calculated cumulative emissions for CH_4_ and N_2_O of each tank by the 100‐year global warming potential of 27 and 273, respectively (IPCC, 2021).

The differences in temperature and some manure characteristics across all tanks at the same sampling date and between different sampling dates were assessed using average ± standard deviation and paired *t*‐tests. The treatment effect on emissions over time was assessed using a repeated measures analysis of variance (ANOVA), and the treatment effect of low, high, and control rates of gypsum additive on CH_4_ and N_2_O emissions was assessed using Tukey's HSD (honestly significant difference). Significant treatment effects were declared when *p* < 0.05.

## 3 RESULTS AND DISCUSSION

### 3.1 Manure and environmental conditions

#### 3.1.1 Manure characteristics

All sample measurements from the beginning of the study (June 6, 2024) were before any treatment additions and will henceforth be referred to as “before treatment.” The pH remained neutral throughout the study period in all the tanks with an average pH of 6.9 ± 0.1 before treatment. At the end of the study (November 24, 2024), there was no notable difference in pH between the treatment tanks but the average pH of 7.4 ± 0.2 was significantly higher (paired *t*‐test, *p* = 0.03) than the average pH before treatment. After the first sample collection in June, the gypsum treatment appeared to increase the settling behavior of manure solids. Due to the different settling behavior of the control and treated tanks in the present study, the sampling strategy of composite analysis of three depths was not effective for representing the TSs in the entire tanks. The implication of this stratification is that comparison of changes in TS, VS, and TN over time could not be done. In the future a different sampling strategy would be needed to integrate across all depths in the stratified manure. As a result of stratification, the manure characteristics from before treatment samples are considered the most homogenous.

Before treatment, the average TS across all tanks was 8.4% ± 0.4 with an average VSs of 6.4% ± 0.4. These values were expected for dairy manure collected from a barn with wood bedding and the values are similar to that seen by Le Riche et al. (2017) who tested manure from a farm that also used wood bedding. Fiber analysis was performed on before treatment samples, which showed similar results from all six tanks: 32% acid detergent fiber, 45% neutral detergent fiber, 11% lignin, 3% crude fat and 4% fat (acid hydrolysis).

The TN includes both organic and inorganic nitrogen present in the slurry and was on average 0.27% ± 0.01 across all tanks before treatment. These values are similar to Le Riche et al. (2017) in the manure samples from a farm that also used wood shavings as bedding material. The total ammoniacal nitrogen (TAN), a subset of TN which refers to the sum of NH_3_ and ammonium ion (NH_4_^+^) present in the manure, was similar across the different treatment tanks (0.14% ± 0.01 before treatment). These results are comparable to previous studies done at the same research site (LeRiche et al., 2017, 2016; Sokolov et al., 2019; Wood et al., 2012). The average TAN at the end of the study was also similar across the different treatment tanks, though slightly lower (0.12% ± 0.02). Although there was less TAN at the end of the study compared to the beginning, the observed decrease was not considered significant (paired *t*‐test, *p* = 0.09). This is an indication that, although there was NH_3_ volatilization, the overall loss was small. Moreover, there was no difference between treatments, indicating that adding gypsum did not change the rate of N loss. In contrast, a study using sulfuric acid treatment on dairy manure at the same site resulted in the TAN higher at the end of the trial period in treated tanks and lower in the control tanks (Sokolov et al., 2019). Therefore, the reduction in NH_3_ volatilization using gypsum was likely modest in comparison to what would have been seen with manure acidification. This suggests that manure acidification is a more effective mitigation method than gypsum alone for reducing NH_3_ volatilization but further research is suggested to explore other trade‐offs.

The FOS‐TAC ratio is a comparison between the fatty acid content (FOS) and the TAC of the manure sample. In batch filled systems, such as slurry storage tanks, it is expected that volatile fatty acids will decline over time by bacterial metabolism as a result of methane production (Lili et al., 2011). According to an Agriculture Biogas Plant assessment (Lili et al., 2011), a FOS‐TAC ratio between 0.3 and 0.4 indicates a stable system leading to maximum CH_4_ production. Before treatment, the average FOS‐TAC ratios were similar in manure samples from all six tanks with the high treatment tanks, low treatment tanks, and control treatment tanks corresponding to ratios of 1.37, 1.44, and 1.44, respectively. At the end of the study, the FOS‐TAC ratio remained steady for the high and low treatment tanks, with average ratios of 1.33 and 1.37, respectively; but showed a considerable decrease in the control tanks with an average ratio of 0.40. These results show that the addition of gypsum to the manure was effective at creating an instable system disrupting CH_4_ production at both the low and high treatment rates. The ORP did not show measurable differences between treatments nor between the beginning and end of the study.

After the addition of gypsum, the treated tanks showed higher measurements of total sulfate and total sulfur from all sampling dates compared to the control tanks (Table 1). The measurements from the tanks with high and low rates of gypsum additive showed a slower decrease in sulfate compared to the control. This outcome was expected since gypsum contains sulfate and therefore provided a saturated environment for sulfate reducing bacteria to outcompete methanogens for substrate thereby reducing CH_4_ emissions (Denier van der Gon et al., 2001). Total sulfur increased after gypsum addition then decreased slowly until the end of the study while remaining higher than the control throughout. Compared to the control, the high treatment rate corresponding to an increase in total sulfur by approximately 38% (fluctuating ± 3% between sampling dates) and the low treatment rate corresponding to an increase in total sulfur by approximately 36% (fluctuating ± 10% between sampling dates). These results are promising regarding the fertilizer value of manure when applied to crops, as they indicate that the addition of gypsum does not cause a substantial increase or decrease in total sulfur content.

**TABLE 1:** Total sulfur and sulfate of manure from all slurry tanks and sampling dates throughout the study period.

Sampling sate	Gypsum addition	Sulfur (ug/g)			Sulfate (ug/g)		
		High rate	Low rate	Control	High rate	Low rate	Control
June 6, 2024	Before	321.68	355.60	333.51	134.09	209.68	148.81
Aug 9, 2024	After	489.80	436.30	302.94	228.03	97.52	41.15
Sept 11, 2024		454.94	495.65	267.05	81.45	85.24	27.55
Oct 31, 2024		430.69	398.73	253.99	72.89	77.77	17.90
Nov 24, 2024		399.07	347.69	257.91	64.63	64.73	52.68

*Note*: The samples from June 6, 2024, were taken before the addition of gypsum. The high rate (7.1 g gypsum L ^−1^ manure) is an average of measurements from Tanks 1 and 4, the low rate (3.2 g gypsum L^−1^ manure) is an average of measurements from Tanks 3 and 5, and the control (0 g gypsum L^−1^ manure) is an average of measurements from Tanks 2 and 6.

#### 3.1.2 Temperature

**FIGURE 1:**
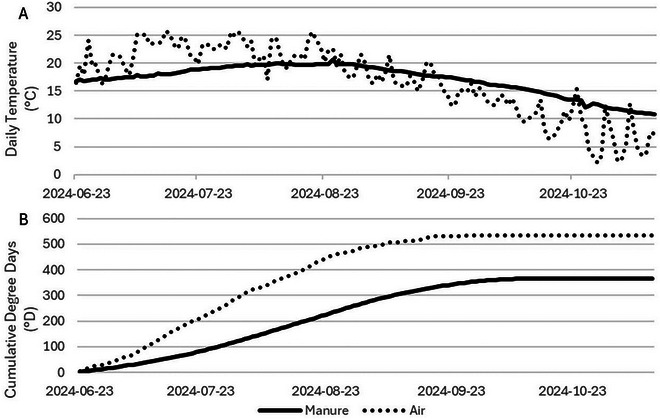
The average daily tank air and manure temperature (A) averaged across all six treatment tanks (°C) and cumulative chamber air and manure temperature (B) across all six treatment tanks in degree days (°D). All air temperature measurements are represented by black dotted lines, while all manure temperature measurements are represented by black smooth lines.

The daily average chamber air temperature ranged from 2°C to 26°C with a mean of 16°C for the entire study period (Figure 1A). The daily average manure temperature ranged from 10°C to 21°C with a mean of 17°C across all six tanks for the entire study period. The average chamber air temperature reached above 25°C in June, July, and August, while manure temperature peaked at 21°C in late‐August. Temperatures steadily decreased until the end of the study period.

The concept of degree days (°D) is a measure of the cumulative temperature deviation from a baseline (Snyder et al., 1999). This concept can be applied to CH_4_ emissions since the rate of methanogenesis has a positive correlation with increasing temperatures (Matos Pereira Lima et al., 2025b). Adopting the baseline of 15°C previously used in lab‐scale gypsum treatment studies, the calculated °D for manure and chamber air were 366 and 536, respectively (Figure 1B). In a laboratory experiment assessing the efficiency of CaSO_4_ (2.04 g L^−1^) at mitigating CH_4_ emissions, the peak mitigation was observed at 300°D for manure kept at a constant temperature of 18°C, slowly decreasing in efficacy thereafter (Matos Pereira Lima et al., 2025b). Since the manure in this study had a total of 366 °D, it follows that the low rate maintained high efficacy for the entire 143 day period. However, if this study were repeated at a warmer location with higher manure temperatures, the low rate of gypsum (3.2 g L^−1^) would probably have lower overall efficacy.

### 3.2 Greenhouse gas emissions

#### 3.2.1 Methane

**FIGURE 2:**
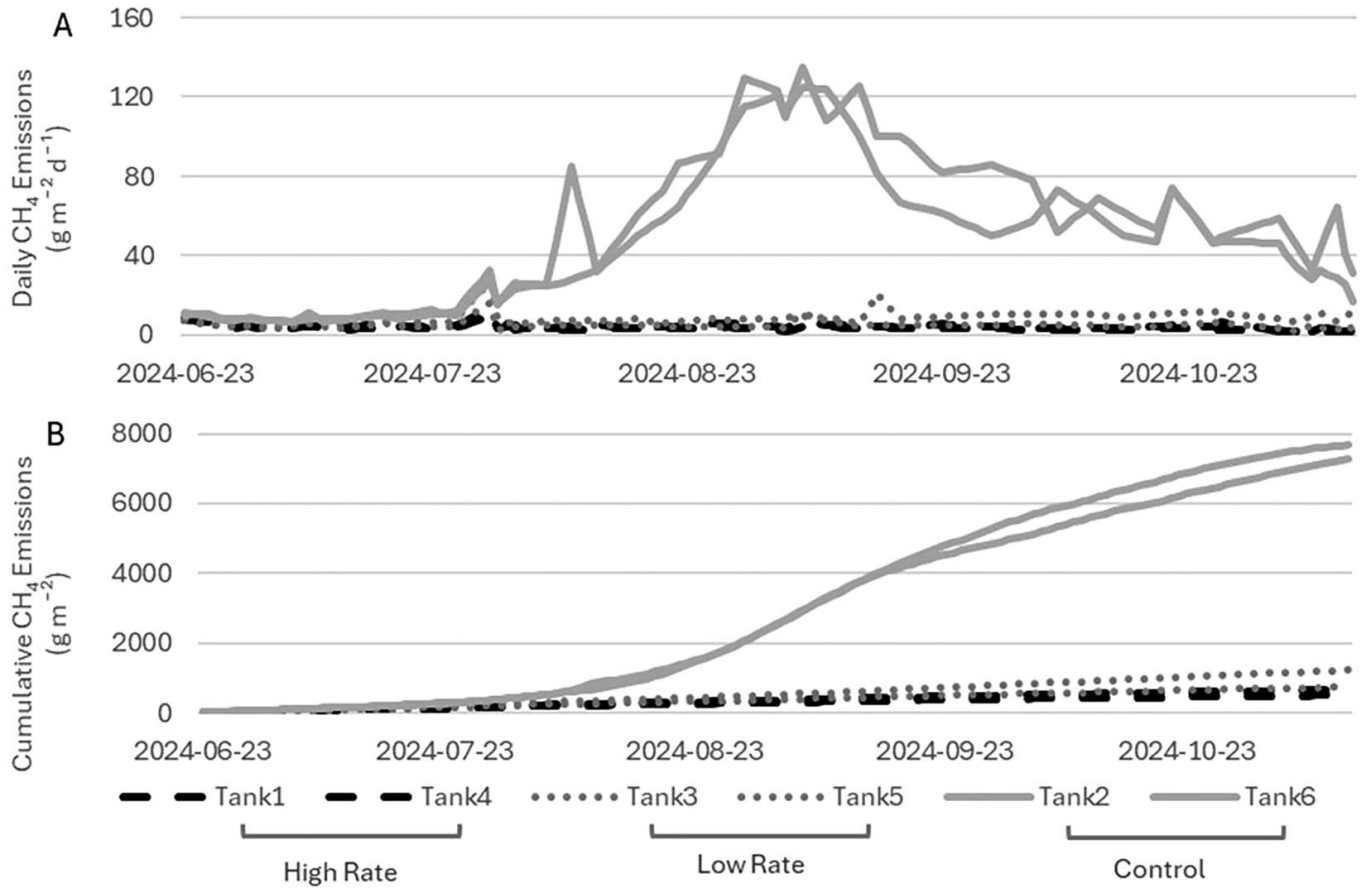
Daily methane (CH_4_) emissions over the 143‐day study period (June 23‐November 12, 2024) for each tank (low rate: 3.30 g L^−1^, high rate: 7.26 g L^−1^, and control: 0 g L^−1^) (A) and cumulative CH_4_ emissions for each tank (B).

Daily average CH_4_ fluxes were 52.29, 6.89, and 4.07 g m^−2^ day^−1^ (or 1.36, 0.18, and 0.11 g m^−3^ h^−1^) for the control, low, and high treatments, respectively (Figure 2A). The control tanks exhibited a lag of 55 days, after which emissions increased linearly until reaching maximum emissions between 86 and 93 days, followed by a slow decline until the end of the study. These results from the control follow an emission curve similar to previous research at the same site (Sokolov et al., 2019; VanderZaag et al., 2010; Wood et al., 2012). Average CH_4_ flux from the control in this warm‐season study was higher than the mean but lower than the maximum of a global review of emissions for cattle slurry stored in tanks (Kupper et al., 2020).

Gypsum‐treated tanks did not exhibit an emission curve similar to the control tanks, nor did they appear to break out of the lag phase. There were brief emission spikes on day 53 for all treatment tanks, and another emission spike was seen on day 102 for one of the low treatment tanks. Previous research has shown that periodic spikes may occur or not occur in all treatment tanks at the same time (VanderZaag et al., 2010). Methane emissions significantly differed over time for all three rates of gypsum additive (repeated measures ANOVA, *p* < 0.001). As mentioned previously, the ideal range of FOS‐TAC ratios for maximum methane production is 0.3–0.4 (Lili et al., 2011). The control tanks maintained ratios within this ideal range, whereas the treated tanks did not—an outcome reflected in the corresponding CH_4_ emission measurements. In situations where CH_4_ emissions cannot be measured, the FOS‐TAC ratio, a simple laboratory test, could be a useful alternative as an indicator of the health of the microbial community.

Cumulative emissions for the 143‐days study period were 7478, 985, and 582 g m^−2^ (or 4674, 616, and 364 g m^−3^) for the control, low, and high rates, respectively (Figure 2B). The cumulative emissions for the control in this study fall within the range of cumulative emissions from the control in previous research at the same research site (2400–13,000 g m^−2^) (LeRiche et al., 2017, 2016; Sokolov et al., 2021, 2019; VanderZaag et al., 2010; Wood et al., 2012).

Compared with the control, cumulative emissions were significantly reduced by 92% and 87% in the high (Tukey's HSD, *p* < 0.001) and low (Tukey's HSD, *p* < 0.001) treatments, respectively. Emissions of CH_4_ from the low and high treatment tanks did not differ significantly from each other (Tukey's HSD, *p* = 0.66). The MCF of the control tanks was on average 0.44 (44%), which is similar to previous studies during the warm season at the same research site (LeRiche et al., 2017; Sokolov et al., 2021). The MCF values for the high‐treatment tanks and low‐treatment tanks were on average 0.03 (3%) and 0.06 (6%), respectively. The average MCF value of the high treatment tanks is similar to the MCF values of manure treated with sulfuric acid at the same research site, where the medium and low pH treatments resulted in MCF values of 0.019 and 0.016, respectively (Sokolov et al., 2019). This is an indication that gypsum treatment could be an option for farmers to greatly reduce CH_4_ emissions from slurry storage.

#### 3.2.2 Nitrous oxide

**FIGURE 3:**
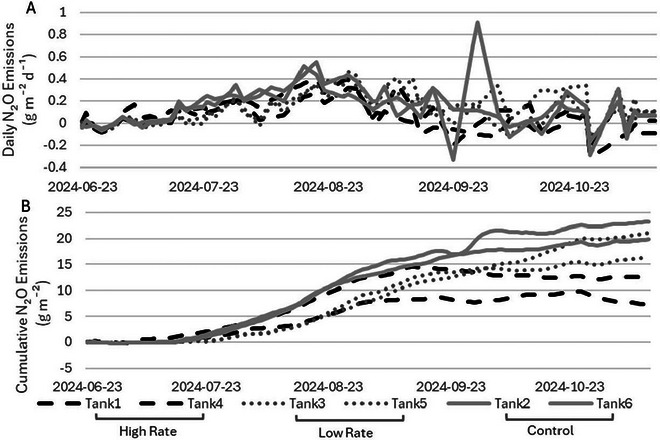
Daily nitrous oxide (N_2_O) emissions over the 143‐day study period (June 23‐November 12, 2024) for each tank (low rate: 3.30 g L^−1^, high rate: 7.26 g L^−1^, and control: 0 g L^−1^) (A) and cumulative N_2_O emissions for each tank (B).

Cumulative N_2_O emissions for the 143‐days study period were on average 22, 19, and 10 g m^−2^ for the control, low, and high treatment tanks, respectively (Figure 3B). The cumulative emissions of the control fall above the range seen in the controls from previous research at the same site (1.27–15 g m^−2^) for a similar seasonal period (LeRiche et al., 2017, 2016; Sokolov et al., 2021, 2019; VanderZaag et al., 2010; Wood et al., 2012). The daily average N_2_O flux was 0.15, 0.13, and 0.07 g m^−2^ day^−1^ (or 0.006, 0.005, and 0.003 g m^−2^ h^−1^) for control, low, and high treatment tanks, respectively (Figure 3A). Compared to a global review of cattle slurry emissions, the N_2_O fluxes are in the highest quartile (Kupper et al., 2020). However, the relatively high N_2_O emissions have a low contribution to the overall footprint of GHG emissions from liquid manure (refer to Section 3.2.3), and we have more uncertainty in our N_2_O emissions given the small trace gas concentrations. Neither the low nor high gypsum treatment rates led to significant change in N_2_O emissions compared to the control (*p*‐value 0.75 and 0.08 for the low and high rates, respectively). It is important to note, however, that treatment of manure with gypsum did not lead to increased N_2_O emissions compared to the control.

**FIGURE 4:**
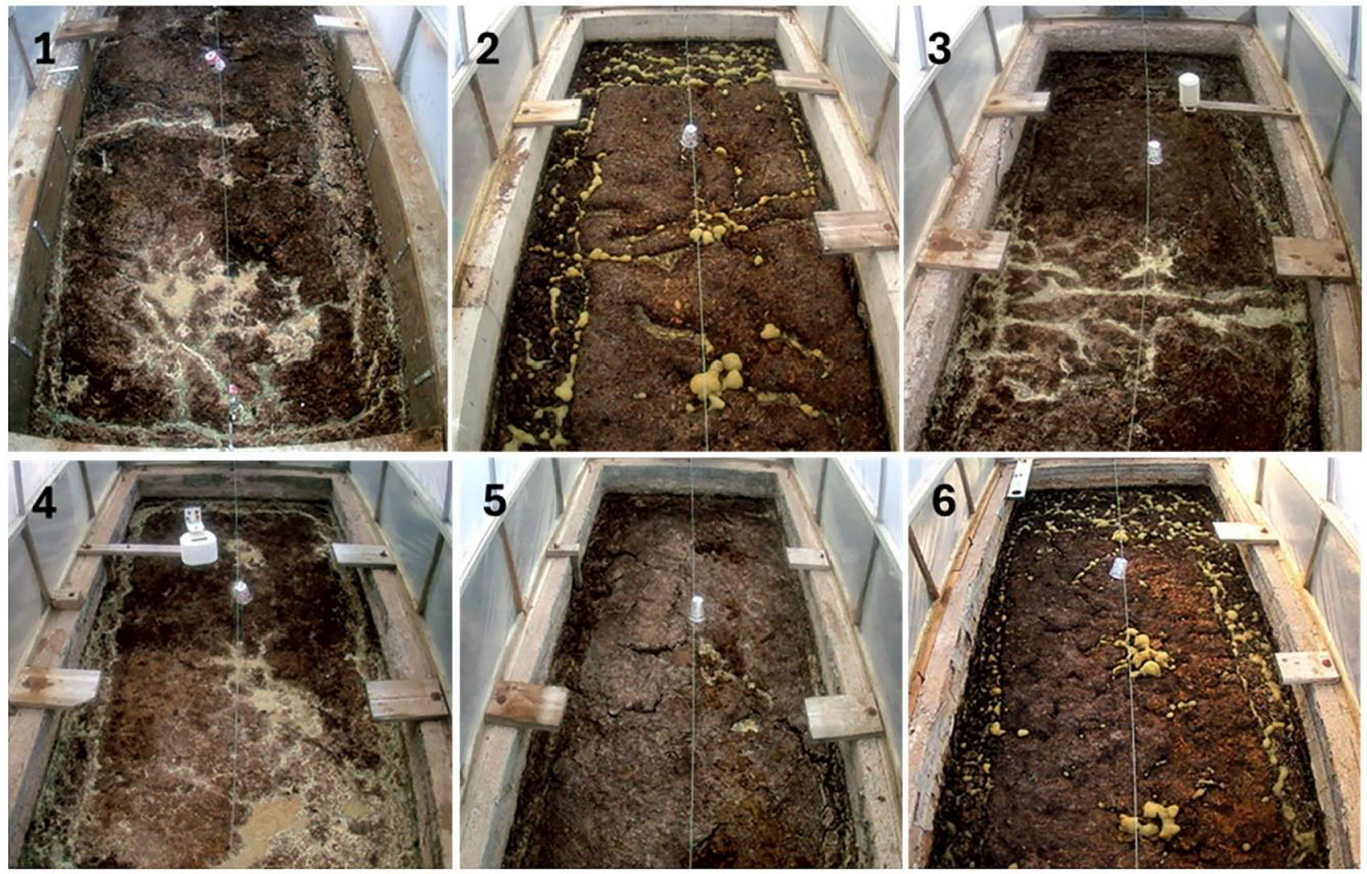
Photographs from a time‐lapse series taken within each tank on September 15, 2024 at 8 a.m. Tanks 1 and 4 represent the high rate of gypsum (7.26 g L^−1^), Tanks 3 and 5 represent the low rate of gypsum (3.30 g L^−1^), and Tanks 2 and 6 represent the control tanks (0 g L^−1^).

The greater emissions of N_2_O compared to previous studies at the same site may be due to the high TS content (>8%), which has been hypothesized to lead to drier crusts on the surface of the slurries, providing an environment well suited to nitrification‐denitrification (Wood et al., 2012). Photos taken within each tank at the same date and time show the difference in crust depending on the treatment rate (Figure 4). The photos show bubbles at the surface of the control tanks, which is an indicator of CH_4_ rising to the surface. Less CH_4_ within the manure means there would be less solids rising with the CH_4_ bubbles, leading to less surface crust formation and therefore less N_2_O production. This can be seen in the photos of the tanks with gypsum treatment, which appear to have less surface crust and lack visible bubbles at the surface.

#### 3.2.3 CO_2_‐e emissions

**FIGURE 5:**
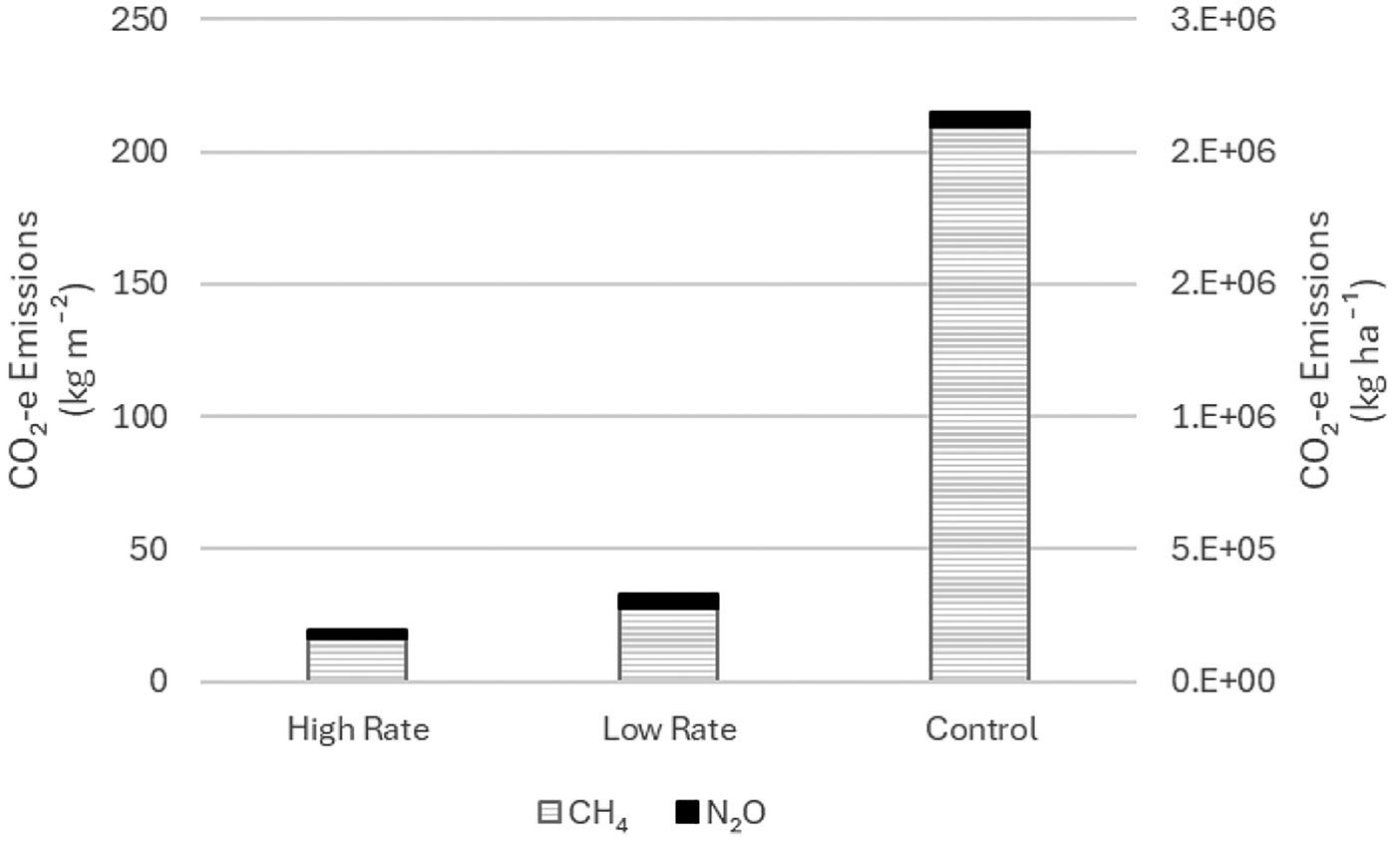
Total greenhouse gas (GHG) emissions (kg m^−2^ and kg ha^−1^) over the 143‐days study period (June 23‐November 12, 2024) in CO_2_‐e emissions for CH_4_ and N_2_O for the control (0 g L^−1^), high rate (7.1 g L^−1^), and low rate (3.2 g L^−1^) gypsum additions to manure.

Overall, the treatment of manure with low and high rates of gypsum additive reduced total CO_2_‐e emissions by 85% and 91% (Figure 5) in comparison to the control emissions, respectively. For the low and high treatment rates, N_2_O emissions equated to only about 14% of total CO_2_‐e emissions, and for the control, N_2_O emissions equated to only about 3% of the total. In our study, the remainder of measured emissions was from the production of CH_4_. This is consistent with previous studies at the same research site reporting that the majority of GHG emissions were from CH_4_ (Le Riche et al., 2016; Sokolov et al., 2019; VanderZaag et al., 2010). Therefore, a reduction in CH_4_ emissions equates to a reduction in overall GHG emissions.

#### 3.2.4 Hydrogen sulfide

H_2_S is not a GHG; rather, it can be toxic if its concentration is above a threshold. Thus, H_2_S was sampled on 1 day in the summer when manure temperature and CH_4_ emissions from the control were near their peak. No H_2_S was detected coming from any of the tanks.

A previous study found farmers that used gypsum bedding had higher risk of elevated downwind concentration of H_2_S during manure agitation (Fabian‐Wheeler et al., 2017). However, the link between gypsum and H_2_S is complex. For example, air samples taken downwind from one farm using gypsum bedding showed no detectable H_2_S during agitation. The amount of gypsum added as bedding in the farms studied by Fabian‐Wheeler et al. (2017) was reported to be from 0.3 to 7.4 lbs cow^−1^ day^−1^ (136–3357 g cow^−1^ day^−1^). Both rates of gypsum used in the present study are at the low end of the range observed by Fabian‐Wheeler et al. (2017) (0.4–0.9 lbs cow^−1^ day^−1^ or 192–429 g cow^−1^ day^−1^). Nevertheless, safety recommendations made by Fabian‐Wheeler et al. (2017) should be taken into consideration when using gypsum at farm scale. For example, operators in enclosed tractor cabs and well away from the downwind rim of the manure storage are at lower risk of exposure to elevated H_2_S during agitation.

### 3.3 On‐farm considerations of handling and cost

Farmers in Canada and many other jurisdictions are not mandated to reduce their carbon footprint; therefore. any adoption of mitigation measures is voluntary. Previous studies into chemical additive mitigation strategies have shown large GHG emission reductions using H_2_SO_4_ (Ambrose et al., 2023; Matos Pereira Lima et al., 2025a; Sokolov et al., 2019). However, H_2_SO_4_ is a highly corrosive chemical necessitating additional safety measures before handling and on‐farm application. This could lead to farmer hesitation.

Based on the results of this study, gypsum addition to slurry storage tanks could be an option for farmers to greatly reduce CH_4_ emissions. Gypsum is simple to handle, as it is not considered a WHMIS‐controlled product and does not require any personal precautions, protective equipment, or emergency procedures (Mosher Limestone Company Limited, 2018). Moreover, in many instances it can be purchased locally either from a mine, as was the case in this study, or potentially recycled gypsum from construction and demolition projects. Additional research is needed to determine on‐farm application rates and efficacy as well as the cost to farmers. As a rough estimate, considering the farm where the manure was obtained has a manure storage volume in late summer of ∼3000 m^3^. The gypsum addition using the low rate of 3.2 g L^−1^ translates to ∼9.6 tonnes and the high rate of 7.1 g L^−1^ translates to ∼21.3 tonnes. Using the price for bulk gypsum at the local quarry from which the gypsum was purchased, the annual cost would be ∼$1373 CAD and $3046 CAD for the low and high rates, respectively. With continuing research into adding gypsum to manure storage systems, there's potential to establish a mechanism to receive payment for reduction in methane emissions through the recently developed Federal Offset Protocol for Reducing Manure Methane Emissions in Canada (the current protocol includes sulfuric acid additives as an eligible mitigation method) (ECCC, 2025). As previously mentioned, farmers will often use their stored manure as a crop fertilizer, sometimes requiring supplemental fertilizers to increase various minerals and nutrients, such as sulfur content. As the manure with the gypsum additive had a higher sulfur content, this could lead to cost savings by requiring less supplemental fertilizer to be applied to meet crop demand.

## 4 CONCLUSION

This pilot‐scale study investigated the benefit of adding gypsum to dairy manure/slurry in reducing CH_4_ and N_2_O emissions. The results showed that both the low rate (3.2 g L^−1^) and high rate (7.1 g L^−1^) of gypsum significantly reduced CH_4_ emissions by 87% and 92%, respectively, compared to the control (no gypsum). Emissions of N_2_O were not significantly changed with the gypsum additive. These findings contribute to the initiative of the dairy industry to reduce the carbon footprint of milk. The promising results from a pilot‐scale research facility suggest that further large‐scale research is needed to evaluate the effectiveness of gypsum on farms. It is important to recognize that a full life cycle analysis is required to also explore any synergies or tradeoffs associated with the field application of gypsum‐amended slurry.

## AUTHOR CONTRIBUTIONS

**Emma Tomalty**: Data curation; formal analysis; validation; visualization; writing—original draft; writing—review and editing. **Mélodie Laniel**: Data curation; formal analysis; investigation; methodology. **Vyncent Leblanc**: Data curation; formal analysis; software. **Ulrica McKim**: Data curation; formal analysis; investigation; methodology. **Sandra Yanni**: Methodology; resources; supervision; writing—review and editing. **Patricia Moher Copa**: Data curation; project administration; resources. **John McCabe**: Data curation; investigation; methodology; resources. **Robert Gordon**: Conceptualization; funding acquisition; supervision; writing—review and editing. **Andrew VanderZaag**: Conceptualization; funding acquisition; methodology; project administration; resources; supervision; validation; visualization; writing—review and editing.

## CONFLICT OF INTEREST STATEMENT

The authors declare no conflicts of interest.

## Data Availability

Datasets are available from the corresponding author upon reasonable request.

## References

[jeq270112-bib-0001] Ambrose, H. W. , Dalby, F. R. , Feilberg, A. , & Kofoed, M. V. W. (2023). Additives and methods for the mitigation of methane emission from stored liquid manure. Biosystems Engineering, 229, 209–245. 10.1016/j.biosystemseng.2023.03.015

[jeq270112-bib-0002] Amon, B. , Kryvoruchko, V. , Moitzi, G. , & Amon, T. (2006). Greenhouse gas and ammonia emission abatement by slurry treatment. International Congress Series, 1293, 295–298. 10.1016/j.ics.2006.01.069

[jeq270112-bib-0003] Baldé, H. , VanderZaag, A. C. , Burtt, S. D. , Gordon, R. J. , & Desjardins, R. L. (2016). Does fall removal of the dairy manure sludge in a storage tank reduce subsequent methane emissions? Journal of Environmental Quality, 45, 2038–2043. 10.2134/jeq2016.03.0083 27898776

[jeq270112-bib-0004] Berg, W. , & Model, A. (2008). Gypsum reduces methane emission during the storage of pig slurry. Australian Journal of Experimental Agriculture, 48, 96–98. 10.1071/EA07226

[jeq270112-bib-0005] Cael, B. B. , & Goodwin, P. A. (2023). Global methane pledge versus carbon dioxide emission reduction. Environmental Research Letters, 18, 104015. 10.1088/1748-9326/acf8dd

[jeq270112-bib-0006] Cárdenas, A. , Ammon, C. , Schumacher, B. , Stinner, W. , Herrmann, C. , Schneider, M. , Weinrich, S. , Fischer, P. , Amon, T. , & Amon, B. (2021). Methane emissions from the storage of liquid dairy manure: Influences of season, temperature and storage duration. Waste Management, 121, 393–402. 10.1016/j.wasman.2020.12.026 33445112

[jeq270112-bib-0007] Crippa, M. , Guizzardi, D. , Solazzo, E. , Muntean, M. , Schaaf, E. , Monforti‐Ferrario, F. , Banja, M. , Olivier, J. G. J. , Grassi, G. , Rossi, S. , & Vignati, E. (2021). GHG emissions of all world countries ‐ 2021 Report, EUR 20821 EN. Publications Office of the European Union. 10.2760/173513

[jeq270112-bib-0008] Dairy Farmers of Canada . (2024). How we're reducing emissions . https://dairyfarmersofcanada.ca/en/sustainability/emissions

[jeq270112-bib-0009] Dalby, F. R. , Hafner, S. D. , Petersen, S. O. , VanderZaag, A. C. , Habtewold, J. , Dunfield, K. , Chantigny, M. H. , & Sommer, S. G. (2021). Understanding methane emission from stored animal manure: A review to guide model development. Journal of Environmental Quality, 50, 817–835. 10.1002/jeq2.20252 34021608

[jeq270112-bib-0010] Denier van der Gon, H. A. , Van Bodegom, P. M. , Wassmann, R. , Lantin, R. S. , & Metra‐Corton, T. M. (2001). Sulfate‐containing amendments to reduce methane emissions from rice fields: Mechanisms, effectiveness and costs. Mitigation and Adaptation Strategies for Global Change, 6, 71–89. 10.1023/A:1011380916490

[jeq270112-bib-0011] Environment and Climate Change Canada . (2024). Canadian environmental sustainability indicators: Greenhouse gas emissions . www.canada.ca/en/environment‐climate‐change/services/environmental‐indicators/greenhouse‐gasemissions.html

[jeq270112-bib-0012] Environment and Climate Change Canada . (2025). Reducing manure methane emissions federal offset protocol: Public consultation draft . https://www.canada.ca/en/environment‐climate‐change/services/climate‐change/pricing‐pollution‐how‐it‐will‐work/output‐based‐pricing‐system/federal‐greenhouse‐gas‐offset‐system/protocols/reducing‐manure‐methane‐emissions/draft.html

[jeq270112-bib-0013] Eriksen, J. , Andersen, A. J. , Poulsen, H. V. , Adamsen, A. P. S. , & Petersen, S. O. (2012). Sulfur turnover and emissions during storage of cattle slurry: Effects of acidification and sulfur addition. Journal of Environmental Quality, 41, 1633–1641. 10.2134/jeq2012.0012 23099955

[jeq270112-bib-0014] Fabian‐Wheeler, E. E. , Hile, M. L. , Murphy, D. J. , Hill, D. E. , Meinen, R. , Brandt, R. C. , Elliott, H. A. , & Hofstetter, D. (2017). Operator exposure to hydrogen sulfide from dairy manure storages containing gypsum bedding. Journal of Agriculture Safety and Health, 23(1), 9–22. 10.13031/jash.11563 29140615

[jeq270112-bib-0049] FAO . (2022). *Methane emissions in livestock and rice systems – Sources, quantification, mitigation and metrics* (Draft for public review). Livestock Environmental Assessment and Performance (LEAP) Partnership. https://openknowledge.fao.org/server/api/core/bitstreams/d1fb956a-1427-4d30-911e-703608d9445b/content

[jeq270112-bib-0015] Filonchyk, M. , Peterson, M. P. , Zhang, L. , Hurynovich, V. , & He, Y. (2024). Greenhouse gases emissions and global climate change: Examining the influence of CO_2_, CH_4_, and N_2_O. Science and the Total Environment, 935, 173359. 10.1016/j.scitotenv.2024.173359 38768722

[jeq270112-bib-0016] Finzi, A. , Vazifehkhoran, A. H. , Dinuccio, E. , Ambrosini, R. , & Provolo, G. (2024). Acidification of livestock slurry and digestate to reduce NH_3_ emissions: Predicting needed H_2_SO_4_ dosage and pH trends over time based on their chemical‐physical composition. Biosystems Engineering, 240, 1–13. 10.1016/j.biosystemseng.2024.02.012

[jeq270112-bib-0017] Flotats, X. , Foged, L. H. , Blasi, A. B. , Palatsi, J. , Magri, A. , & Schelde, K. M. (2011). Manure processing technologies. Technical report no. II concerning Manure Processing Activities in Europe to the European Commission, *Directorate‐General Environment* . European Union. https://op.europa.eu/en/publication-detail/-/publication/583aacc3-8e66-40d8-8828-47f8cd4262d7

[jeq270112-bib-0018] Hjorth, M. , Christensen, K. V. , Christensen, M. L. , & Sommer, S. G. (2010). Solid‐liquid separation of animal slurry in theory and practice. A review. Agronomy for Sustainable Development, 30, 153–180. 10.1051/agro/2009010

[jeq270112-bib-0019] Holly, M. A. , Larson, R. A. , & Powell, J. M. (2015). Gas reduction benefits from storage to application of anaerobic digestion and solid‐liquid separation of dairy manure [Paper presentation]. 2015 ASABE Annual International Meeting, New Orleans, Louisiana, United States. 10.13031/aim.20152190156

[jeq270112-bib-0020] Im, S. , Petersen, S. O. , Lee, D. , & Kim, D.‐H. (2020). Effects of storage temperature on CH_4_ emissions from cattle manure and subsequent biogas production potential. Waste Management, 101, 35–43. 10.1016/j.wasman.2019.09.036 31586875

[jeq270112-bib-0021] IPCC . (2019). Emissions from livestock and manure management. In 2019 Refinement to the 2006 IPCC guidelines for national greenhouse gas inventories (Vol. 4, p. 209). Agriculture, Forestry and Other Land Use. https://www.ipcc‐nggip.iges.or.jp/public/2019rf/index.html

[jeq270112-bib-0022] IPCC . (2021). Summary for policymakers (AR6) Climate Change 2021: The Physical Science Basis: Contribution of working group I to the sixth assessment report of the Intergovernmental Panel on Climate Change . IPCC.

[jeq270112-bib-0023] Kai, P. , Pedersen, P. , Jensen, J. E. , Hansen, M. N. , & Sommer, S. G. (2008). A whole‐farm assessment of the efficacy of slurry acidification in reducing ammonia emissions. European Journal of Agronomy, 28, 148–154. 10.1016/j.eja.2007.06.004

[jeq270112-bib-0024] Kupper, T. , Hani, C. , Neftel, A. , Kincaid, C. , Buhler, M. , Amon, B. , & VanderZaag, A. (2020). Ammonia and greenhouse gas emissions from slurry storage—A review. Agriculture, Ecosystems & Environment, 300, 106963. 10.1016/j.agee.2020.106963

[jeq270112-bib-0025] Lemes, Y. M. , Garcia, P. , Nyord, T. , Fielberg, A. , & Kamp, J. N. (2022). Full‐scale investigation of methane and ammonia mitigation by early single‐dose slurry storage acidification. ACS Agricultural Science & Technology, 2(6), 1196–1205. 10.1021/acsagscitech.2c00172

[jeq270112-bib-0026] Le Riche, E. L. , VanderZaag, A. C. , Wagner‐Riddle, C. , Dunfield, K. , Sokolov, V. K. , & Gordon, R. (2017). Do volatile solids from bedding materials increase greenhouse gas emissions for stored dairy manure? Canadian Journal of Soil Science, 97, 512–521. 10.1139/cjss-2016-0119

[jeq270112-bib-0027] Le Riche, E. L. , VanderZaag, A. C. , Wood, J. D. , Wagner‐Riddle, C. , Dunfield, K. , Ngwabie, N. M. , McCabe, J. , & Gordon, R. J. (2016). Greenhouse gas emissions from stored dairy slurry from multiple farms. Journal of Environmental Quality, 45, 1822–1828. 10.2134/jeq2016.04.0122 27898800

[jeq270112-bib-0028] Lili, M. , Biro, G. , Sulyok, E. , Petis, M. , Borbely, J. , & Tamas, J. (2011). Novel approach on the basis of FOS/TAC method. Analele Universitatii din Oradea, Fascicula Protectia Mediului, 17, 713–718.

[jeq270112-bib-0029] Livingston, G. P. , & Hutchinson, G. L. (1995). Enclosure‐based measurement of trace gas exchange: Applications and sources of error. In P. A. Matson & R. C. Harriss (Eds.), Biogenic trace Gases: Measuring emissions from soil and water (pp. 14–51). Blackwell Synergy.

[jeq270112-bib-0030] Malley, C. S. , Borgford‐Parnell, N. , Haeussling, S. , Howard, I. C. , Lefevre, E. N. , & Kuylenstierna, J. C. I. (2023). A roadmap to achieve the global methane pledge. Environmental Research: Climate, 2, 011003. 10.1088/2752-5295/acb4b4

[jeq270112-bib-0031] Matos Pereira Lima, F. , Laniel, M. , Baldé, H. , Gordon, R. , & VanderZaag, A. (2025a). Methane emission reduction by adding sulfate to liquid dairy manure. Journal of Environmental Quality, 54, 349–358. 10.1002/jeq2.70002 39957420 PMC11893280

[jeq270112-bib-0032] Matos Pereira Lima, F. , Laniel, M. , Baldé, H. , Gordon, R. , & VanderZaag, A. (2025b). Sulfate additives cut methane emissions more effectively at lower manure temperatures. ACS Agricultural Science & Technology. 10.1021/acsagscitech.4c00659

[jeq270112-bib-0033] Mosher Limestone Company Limited . (2018). Material safety data sheet: Agricultural gypsum, industrial gypsum powder . Mosher Limestone Company Limited.

[jeq270112-bib-0034] Nisbet, E. G. , Manning, M. R. , Lowry, D. , Fisher, R. E. , Lan, X. , Michel, S. E. , France, J. L. , Nisbet, R. E. R. , Bakkaloglu, S. , Leitner, S. M. , Brooke, C. , Rockmann, T. , Allen, G. , Denier Van der Gon, H. A. C. , Merbold, L. , Scheutz, C. , Maisch, C. W. , Nisbet‐Jones, P. B. R. , Alshalan, A. , … Dlugokencky, E. J. (2025). Practical paths towards quantifying and mitigating agricultural methane emissions. Proceedings of the Royal Society A: Mathematical, Physical and Engineering Sciences, 481, 20240390. 10.1098/rspa.2024.0390

[jeq270112-bib-0035] Osuwu‐Twum, M. Y. , Kelleghan, D. , Gleasure, G. , Connolly, S. , Forrestal, P. , Lanigan, G. J. , Richards, K. G. , & Krol, D. J. (2024). Mitigation of ammonia and methane emissions with manure amendments during storage of cattle slurry. Waste Management & Research, 43(4), 568–579. 10.1177/0734242x241265007 39069727

[jeq270112-bib-0036] Petersen, S. O. , Andersen, A. J. , & Eriksen, J. (2012). Effects of cattle slurry acidification on ammonia and methane evolution during storage. Journal of Environmental Quality, 41, 88–94. 10.2134/jeq2011.0184 22218177

[jeq270112-bib-0038] Sauvé, C. , Baldé, H. , Rajagopal, R. , & VanderZaag, A. (2025). Methane reductions with gypsum and SOP® Lagoon additives in liquid manure. Frontiers in Climate, 7, 1592677. 10.3389/fckun,2025.10592677

[jeq270112-bib-0039] Snyder, R. L. , Spano, D. , Cesaraccio, C. , & Duce, P. (1999). Determining degree‐day thresholds from field observations. International Journal in Biometerology, 42, 177–182. 10.1007/s004840050102

[jeq270112-bib-0040] Sokolov, V. , Habtewold, J. , VanderZaag, A. , Dunfield, K. , Gregorich, E. , Wagner‐Riddle, C. , Venkiteswaran, J. J. , & Gordon, R. (2021). Response curves for ammonia and methane emissions from stored liquid manure receiving low rates of sulfuric acid. Frontiers in Sustainable Food Systems, 5, 678992. 10.3389/fsufs.2021.678992

[jeq270112-bib-0041] Sokolov, V. , VanderZaag, A. , Habtewold, J. , Dunfield, K. , Wagner‐Riddle, C. , Venkiteswaran, J. J. , & Gordon, R. (2019). Greenhouse gas mitigation through dairy manure acidification. Journal of Environmental Quality, 48(5), 1435–1443. 10.2134/jeq2018.10.0355 31589733

[jeq270112-bib-0042] Intergovernmental Panel on Climate Change (IPCC) . (2013). Climate change 2013: The physical science basis. In T. F. Stocker , D. Qin , G.‐K. Plattner , M. Tignor , S. K. Allen , J. Boschung , A. Nauels , Y. Xia , V. Bex , & P. M. Midgley (Eds.), Contribution of working group I to the fifth assessment report of the intergovernmental panel on climate change (p. 1535). Cambridge University Press.

[jeq270112-bib-0043] United Nations . (2015). Paris agreement . https://unfccc.int/process‐and‐meetings/the‐paris‐agreement/the‐paris‐agreement

[jeq270112-bib-0044] VanderZaag, A. C. , Baldé, H. , Crolla, A. , Gordon, R. J. , Ngwabie, M. , Wagner‐Riddle, C. , Desjardins, R. , & MacDonald, J. D. (2018). Potential methane emission reductions for two manure treatment technologies. Environmental Technology, 39(7), 851–858. 10.1080/09593330.2017.1313317 28355494

[jeq270112-bib-0045] VanderZaag, A. C. , Gordon, R. J. , Jamieson, R. C. , Burton, D. L. , & Stratton, G. W. (2010). Permeable synthetic covers for controlling emissions from liquid dairy manure. Applied Engineering in Agriculture, 26(2), 287–297. 10.13031/2013.29544

[jeq270112-bib-0046] Vechi, N. T. , Falk, J. M. , Fredenslund, A. M. , Edjabou, M. E. , & Scheutz, C. (2023). Methane emission rates averaged over a year from ten farm‐scale manure storage tanks. Science and the Total Environment, 904, 166610. 10.1016/j.scitotenv.2023.166610 37640081

[jeq270112-bib-0047] Vechi, N. T. , Jensen, N. S. , & Scheutz, C. (2022). Methane emissions from five Danish pig farms: Mitigation strategies and inventory estimated emissions. Journal of Environmental Management, 317, 115319. 10.1016/j.jenvman.2022.115319 35642810

[jeq270112-bib-0048] Wood, J. D. , Gordon, R. J. , Wagner‐Riddle, C. , Dunfield, K. E. , & Madani, A. (2012). Relationships between dairy slurry total solids, gas emissions, and surface crusts. Journal of Environmental Quality, 41, 694–704. 10.2134/jeq2011.0333 22565251

